# High-intensity Interval Training Improves Mitochondrial Function and Suppresses Thrombin Generation in Platelets undergoing Hypoxic Stress

**DOI:** 10.1038/s41598-017-04035-7

**Published:** 2017-06-23

**Authors:** Li-Hua Wu, Shao-Chiang Chang, Tieh-Cheng Fu, Ching-Hui Huang, Jong-Shyan Wang

**Affiliations:** 1 Healthy Aging Research Center, Graduate Institute of Rehabilitation Science, Medical Collage, Chang Gung University, Tao-Yuan, Taiwan; 2 Heart Failure Center, Department of Physical Medicine and Rehabilitation, Chang Gung Memorial Hospital, Keelung, Taiwan; 3 Research Center for Chinese Herbal Medicine, College of Human Ecology, Chang Gung University of Science and Technology, Tao-Yuan, Taiwan

## Abstract

This study elucidates how high-intensity interval training (HIT) and moderate-intensity continuous training (MCT) affect mitochondrial functionality and thrombin generation (TG) in platelets following hypoxic exercise (HE, 100 W under 12% O_2_ for 30 min). Forty-five healthy sedentary males were randomized to engage either HIT (3-minute intervals at 40% and 80%VO_2max_, n = 15) or MCT (sustained 60%VO_2max_, n = 15) for 30 minutes/day, 5 days/week for 6 weeks, or to a control group (CTL, n = 15) that did not received exercise intervention. Before the intervention, HE (i) reduced the ATP-linked O_2_ consumption rate (OCR), the reserve capacity of OCR, and the activities of citrate synthase (CS) and succinate dehydrogenase (SDH), (ii) lowered mitochondrial membrane potential (MP) and elevated matrix oxidant burden (MOB) in platelets, and (iii) enhanced dynamic TG in platelet-rich plasma (PRP), which responses were attenuated by pretreating PRP with oligomycin or rotenone/antimycin A. However, 6-week HIT (i) increased mitochondrial OCR capacity with enhancing the CS and SDH activities and (ii) heightened mitochondrial MP with depressing MOB in platelets following HE, compared to those of MCT and CTL. Moreover, the HIT suppressed the HE-promoted dynamic TG in PRP. Hence, we conclude that the HIT simultaneously improves mitochondrial bioenergetics and suppresses dynamic TG in platelets undergoing hypoxia.

## Introduction

Regular exercise may reduce the risk of major vascular thrombotic events and protect people against cardiovascular diseases^1, 2^. Moreover, high-intensity exercise elicits a greater central hemodynamic adaptation than low and moderate levels of exercise^3^. However physical exercise imposes, paradoxically, both enhancing and suppressing effects on platelet reactivity and coagulation, depending on the type and intensity of exercise^1^. Systemic hypoxia has detrimental effects on the haemostatic system, such as accelerating prothrombinase complex assembly and facilitating thrombin generation (TG)^4, 5^. Pathological investigations have also demonstrated that a hypercoagulable state contributes to increased risks of vascular thrombotic events in patients with episodic hypoxia, such as obstructive sleep apnea^6^ and chronic obstructive pulmonary disease^7^. To the best of our knowledge, what kind of exercise strategy improves aerobic capacity and simultaneously increases the resistance to thrombotic risk provoked by hypoxia has not yet been established.

Increased thrombin activity is an essential pathogenic process of cardiovascular diseases^8^. Functional mitochondria in platelet has demonstrated the direct involvements of the cellular ATP production, redox balance, and thrombogenesis^9, 10^. Platelet activation results in its mitochondrial membrane potential collapse and reactive oxygen species (ROS) production, consequently facilitating the exposure of phosphatidylserine (PS)^10^. The negatively charged PS on platelets can bind coagulant factors VIII (FVIII), Va, and Xa, providing a strongly catalytic surface for the assembly of prothrombinase and tenase, thus triggering TG in blood^10^. Our early study further demonstrated that severe hypoxic exposure (12% O_2_) promoted FVIII-dependent TG by elevating oxidative stress; this hypoxic effect was ameliorated by pretreatment with antioxidant vitamin E^5^. However, a previous study has indicated that exercise training decreased the coagulant factor expression and PS exposure of platelets and the shedding of procoagulant microparticles from platelets, thereby depressing dynamic TG in platelets^11^.

Warm-up exercise (40%VO_2max_) has been shown to reduce high-intensity exercise (80%VO_2max_)-induced risks of inflammatory thrombosis associated with leukocytes and platelets, which is a form of preconditioning^12^. Recently, our investigation revealed that high-intensity interval exercise training (HIT) that consists of alternating mild-(40%VO_2max_) and high-(80%VO_2max_) intensity exercise significantly suppressed neutrophil-promoted blood coagulation by down-regulating expression of procoagulant factors under hypoxic condition^13^. Accordingly, we further hypothesize that HIT effectively reduces platelet-induced TG undergoing hypoxic stress, which is associated with alleviating platelet mitochondrial dysfunction cause by hypoxia.

To answer the abovementioned questions, this study evaluated how two isovolumic exercise regimens [*i*.*e*., HIT (3-minute intervals at 40% and 80%VO_2max_) and moderate-intensity continuous exercise training (MCT, sustained 60%VO_2max_)] for 6 weeks affected (i) mitochondrial oxidative phosphorylation (OXPHOS) and oxidative stress, (ii) mitochondrial biogenesis, and (iii) dynamic TG in platelets following hypoxic exercise (HE, 100 W under 12% O_2_ for 30 min). The aim of the present study is to establish an effective exercise strategy for improving individual aerobic capacity and simultaneously ameliorating the risk of platelet mitochondrial dysfunction and subsequent TG evoked by hypoxic stress.

## Results

### Aerobic fitness

Anthropometric variables did not significantly differ among the three groups at the beginning of the study (Table 1). Both HIT and MCT for 6 weeks lowered HR and systolic blood pressure at rest, as well as, increased work-rate, minute ventilation (V_E_), O_2_ consumption (VO_2_), and CO_2_ production (VCO_2_) at the ventilation threshold and peak performance (Table 1, *P* < 0.05). Moreover, the HIT group exhibited a greater improvement in pulmonary ventilation and aerobic capacity than did the MCT group (Table 1, *P* < 0.05). However, control subjects that did not receive exercise intervention (CTL) for 6 weeks showed no changes in these cardiopulmonary responses to a graded exercise test (GXT) (Table 1).

**Table 1:** The effects of interval and continuous exercise regimens on exercise performance.

	HIT		MCT		CTL	
	Pre	Post	Pre	Post	Pre	Post
***Anthropometric parameters***						
Age (year)	22.2 ± 0.4	—	22.1 ± 0.5	—	21.9 ± 0.7	—
Height (cm)	171.0 ± 1.0	—	172.1 ± 1.2	—	172.2 ± 1.3	—
Weight (kg)	69.7 ± 1.1	70.3 ± 2.1	67.3 ± 1.9	67.0 ± 1.8	67.8 ± 3.1	67.8 ± 3.1
BMI (kg/m^2^)	23.8 ± 0.5	23.4 ± 0.6	22.0 ± 0.5	21.9 ± 0.5	22.3 ± 0.8	22.3 ± 0.8
HR (beats/min)	72 ± 1	68 ± 2^+^	71 ± 2	67 ± 2^+^	73 ± 2	72 ± 3
SBP (mmHg)	120 ± 2	115 ± 2^+^	121 ± 3	116 ± 3^+^	123 ± 2	122 ± 3
DBP (mmHg)	74 ± 2	72 ± 2	74 ± 3	72 ± 2	75 ± 3	74 ± 3
***Ventilation threshold***						
Work-rate (watt)	100 ± 5	156.5 ± 7.5*^+^	99.4 ± 5.7	131.8 ± 6.0*	104.0 ± 6.2	108.1 ± 6.2
HR (beats/min)	133 ± 2	154 ± 2.8*^+^	134 ± 3	146 ± 4.0*	132 ± 4	134 ± 5
$\dot{{\rm{V}}}$_E_ (l/min)	36.2 ± 1.8	53.5 ± 3.0*^+^	36.5 ± 2.1	47.0 ± 2.6*	35.2 ± 4.2	36.5 ± 2.6
$\dot{{\rm{V}}}$O_2_ (ml/min/kg)	16.4 ± 0.7	23.8 ± 1.0*^+^	17.5 ± 0.7	21.2 ± 0.9*	16.9 ± 0.9	17.0 ± 0.8
$\dot{{\rm{V}}}$CO_2_ (ml/min/kg)	16.5 ± 0.7	23.8 ± 1.0*^+^	17.5 ± 0.7	21.4 ± 1.1*	17.0 ± 0.9	17.2 ± 0.9
***Peak performance***						
Work-rate (watt)	184.7 ± 6.3	237.6 ± 7.0*^+^	182.4 ± 5.4	213.5 ± 5.5*	180 ± 7	182 ± 9
HR (beats/min)	194 ± 2	195 ± 2	193 ± 2	194 ± 2	195 ± 3	194 ± 2
$\dot{{\rm{V}}}$_E_ (l/min)	103.3 ± 3.0	133.6 ± 4.2*^+^	98.7 ± 3.5	114.1 ± 3.2*	105.1 ± 4.1	104.2 ± 5.1
$\dot{{\rm{V}}}$O_2_ (ml/min/kg)	31.7 ± 0.5	39.7 ± 0.6*^+^	32.2 ± 0.6	36.2 ± 0.6*	31.4 ± 0.6	31.4 ± 0.6
$\dot{{\rm{V}}}$CO_2_ (ml/min/kg)	38.0 ± 0.6	47.4 ± 0.7^*,+^	38.6 ± 0.8	43.4 ± 0.8*	37.7 ± 0.7	37.5 ± 0.6

Values were mean ± SEM. HIT, high-intensity interval training group; MCT, moderate-intensity continuous training group; CTL, control group; Pre, pre-intervention; Post, post-intervention; BMI, body mass index; $\dot{{\rm{V}}}$_E_, minute ventilation; $\dot{{\rm{V}}}$O_2_, oxygen consumption; $\dot{{\rm{V}}}$CO_2_, carbon dioxide production. *P < 0.05, Pre vs. Post; ^+^ *P* < 0.05, HIT vs. MCT.

### Mitochondrial membrane potential (MP), matrix oxidant burden (MOB), and biogenesis in platelets

**Figure 1:**
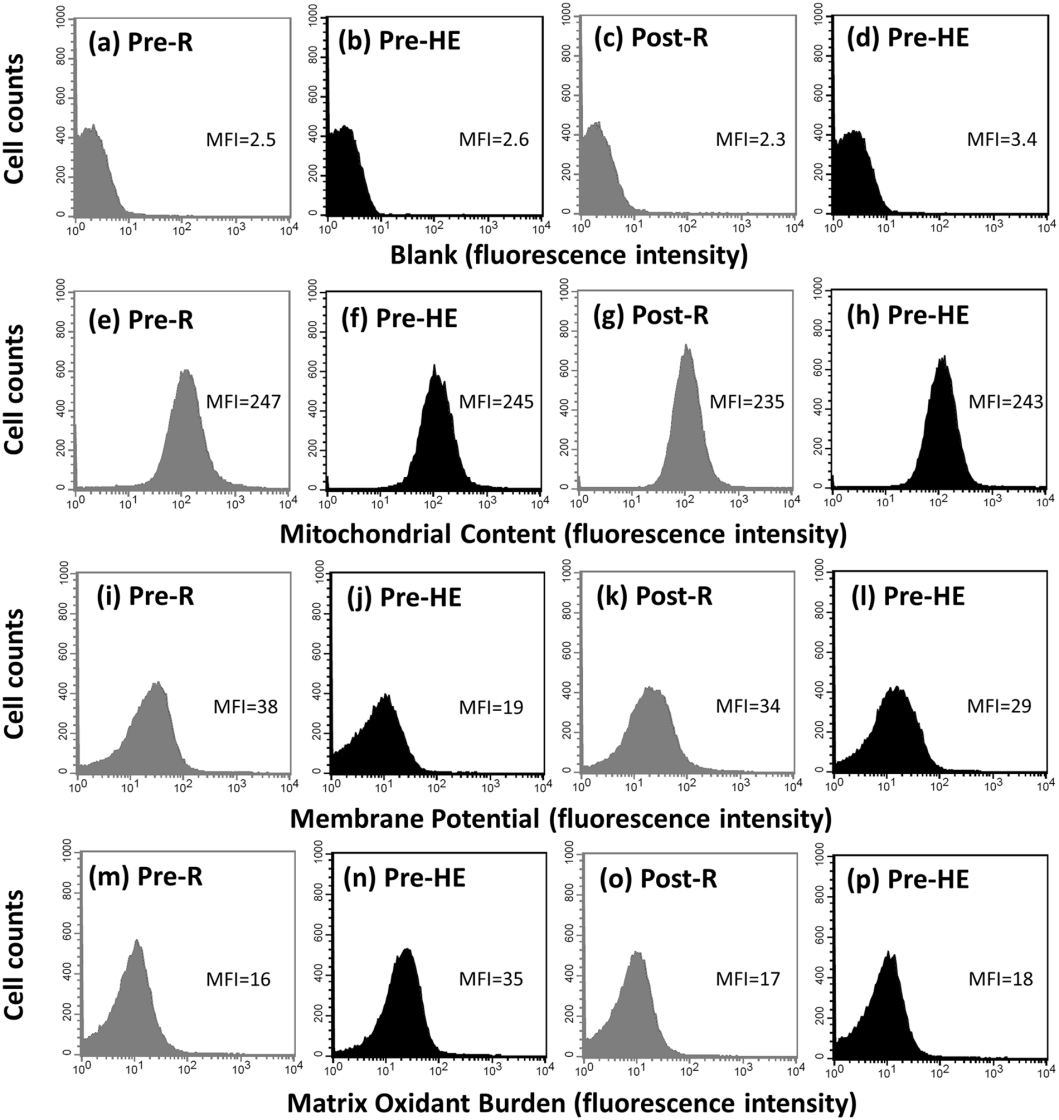
Graph showing the effect of high-intensity interval training (**HIT**) on mitochondrial content, mitochondrial membrane potential, and matrix oxidant burden in platelets using a FACScan flow cytometer. **Pre**, before HIT; **Post**, after HIT; **R**, resting; **HE**, hypoxic (12%O_2_) exercise test. **Blank (a–d)**, platelets without treating fluorescent dye; **Mitochondrial content (e–h)**, platelets stained with MitoTracker Green FM; **Mitochondrial membrane potential (i–l)**, platelet stained with tetramethylrhodamine ethyl ester; **Matrix oxidant burden (m–p)**, platelet stained with MitoSOX Red; **MFI**, mean fluorescence intensity.

**Figure 2:**
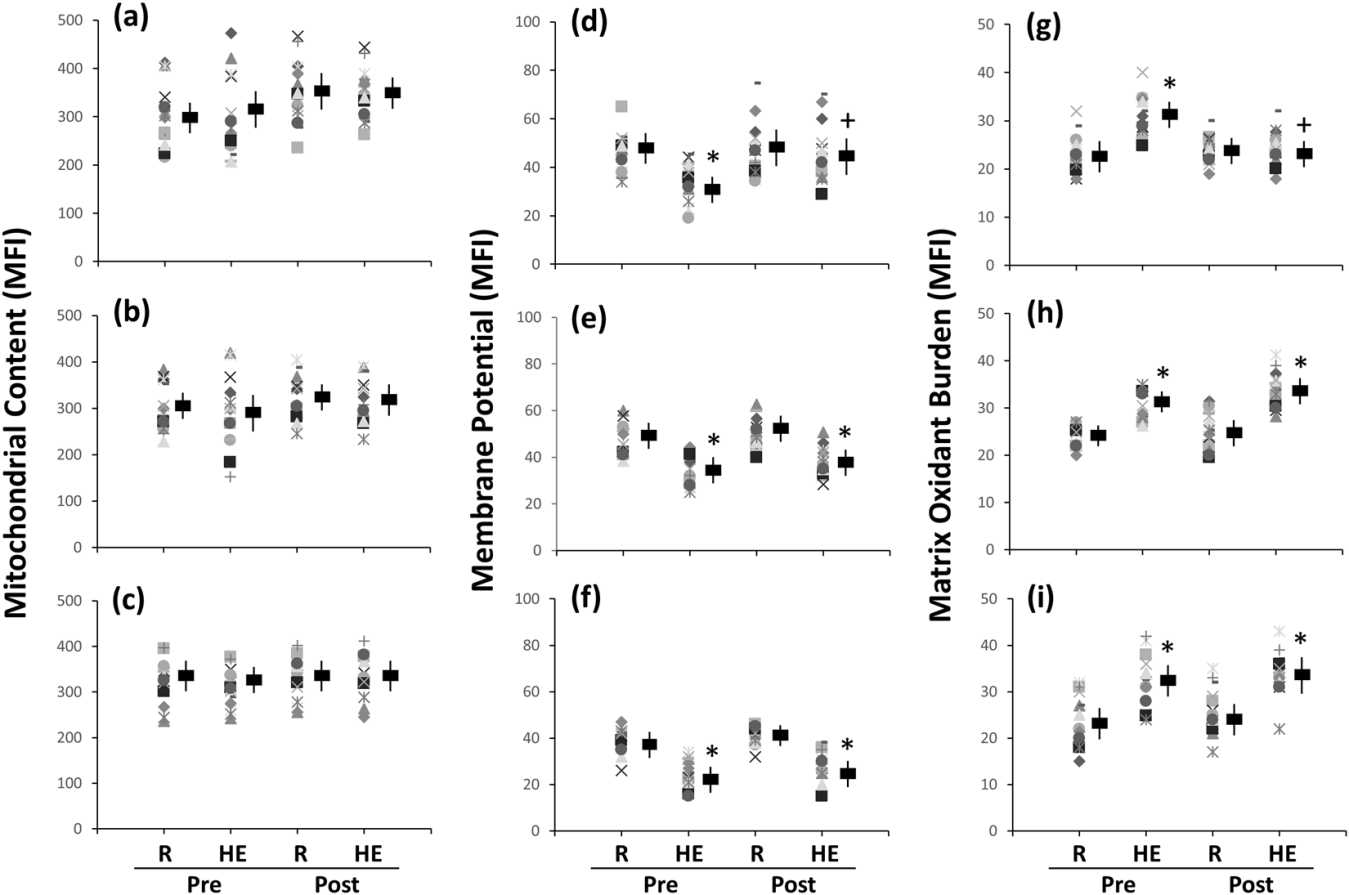
Effects of interval and continuous exercise regimens on **(a–c)** mitochondrial content, **(d–f)** mitochondrial membrane potential, and **(g–i**) matrix oxidant burden in platelets. **HIT**, high-intensity interval training group **(a**,**d**,**g)**; **MCT**, moderate-intensity continuous training **(b**,**e**,**h)**; **CTL**, control group **(c**,**f**,**i)**; **Pre**, pre-intervention; **Post**, post-intervention; **R**, resting; **HE**, hypoxic (12%O_2_) exercise test; **MFI**, mean fluorescence intensity. * *P* < 0.05, **R** vs. **HE**; ^+^ *P* < 0.05, **Pre** vs. **Post**. Values were mean ± SEM.

Acute bout of 12% O_2_ exercise significantly increased platelet counts in blood (*P* < 0.05, Table 2). However, blood platelet counts before or after the HE test remained unchanged following the 6-week intervention with HIT, MCT or CTL (Table 2). Figure 1 shows the analysis of platelet mitochondrial count (Fig. 1e–h), MP (Fig. 1i–l), and MOB (Fig. 1m–p) using a FACScan flow cytometer. Although no changes were observed in the mitochondrial count (Fig. 2a–c), the HE decreased the mitochondrial MP (Fig. 2d–f, *P* < 0.05) and elevated the MOB (Fig. 2g–i, *P* < 0.05) in platelets. After 6 weeks of the intervention, HIT, rather than MCT, inhibited the HE-induced decrease of mitochondrial MP (Fig. 2d, *P* < 0.05) and increase of MOB (Fig. 2g, *P* < 0.05) in platelets. However, no significant changes in the mitochondrial count, MP, and MOB of platelets were observed after CTL for 6 weeks (Fig. 2c,f,i).

**Table 2:** The effects of interval and continuous exercise regimens on platelet count and mitochondrial biogenesis.

		HIT		MCT		CTL	
		Pre	Post	Pre	Post	Pre	Post
***Platelet count (x10*** ^***3***^ ***cells/μl)***							
	R	224 ± 11	216 ± 12	232 ± 10	226 ± 18	234 ± 12	237 ± 9
	HE	264 ± 21*	252 ± 23*	272 ± 16*	266 ± 12*	284 ± 20*	287 ± 17*
***Platelet mitochondrial biogenesis (mean fluorescence intensity)***							
Complex II	R	21.7 ± 2.8	22.1 ± 2.4	22.6 ± 1.9	22.5 ± 2.6	23.5 ± 1.9	23.4 ± 2.5
	HE	21.6 ± 2.3	23.1 ± 2.8	23.0 ± 1.6	23.9 ± 2.5	23.7 ± 2.1	24.3 ± 1.7
Complex IV	R	16.9 ± 2.8	17.0 ± 2.5	16.3 ± 1.7	16.9 ± 2.1	17.4 ± 1.3	16.9 ± 1.5
	HE	16.0 ± 2.7	17.1 ± 1.9	16.7 ± 1.1	16.8 ± 1.4	16.9 ± 1.7	17.0 ± 0.8
Ratio of Complex IV to Complex II							
	R	0.78 ± 0.04	0.77 ± 0.03	0.72 ± 0.05	0.71 ± 0.04	0.74 ± 0.04	0.72 ± 0.03
	HE	0.74 ± 0.06	0.73 ± 0.04	0.73 ± 0.04	0.72 ± 0.05	0.71 ± 0.03	0.70 ± 0.04

Values were mean ± SEM. HIT, high-intensity interval training group; MCT, moderate-intensity continuous training group; CTL, control group; Pre, pre-intervention; Post, post-intervention; R, resting; HE, hypoxic exercise test. *P < 0.05, R vs. HE.

In mitochondrial biogenesis, acute HE did not change the levels of Complex IV and II, as well as, the ratio of Complex IV to II in platelets (Table 2). Furthermore, no significant changes in these mitochondrial biogenetic variables of platelets occurred after 6-week interventions with HIT, MCT, or CTL (Table 2).

### Mitochondrial respiration of platelets

**Figure 3:**
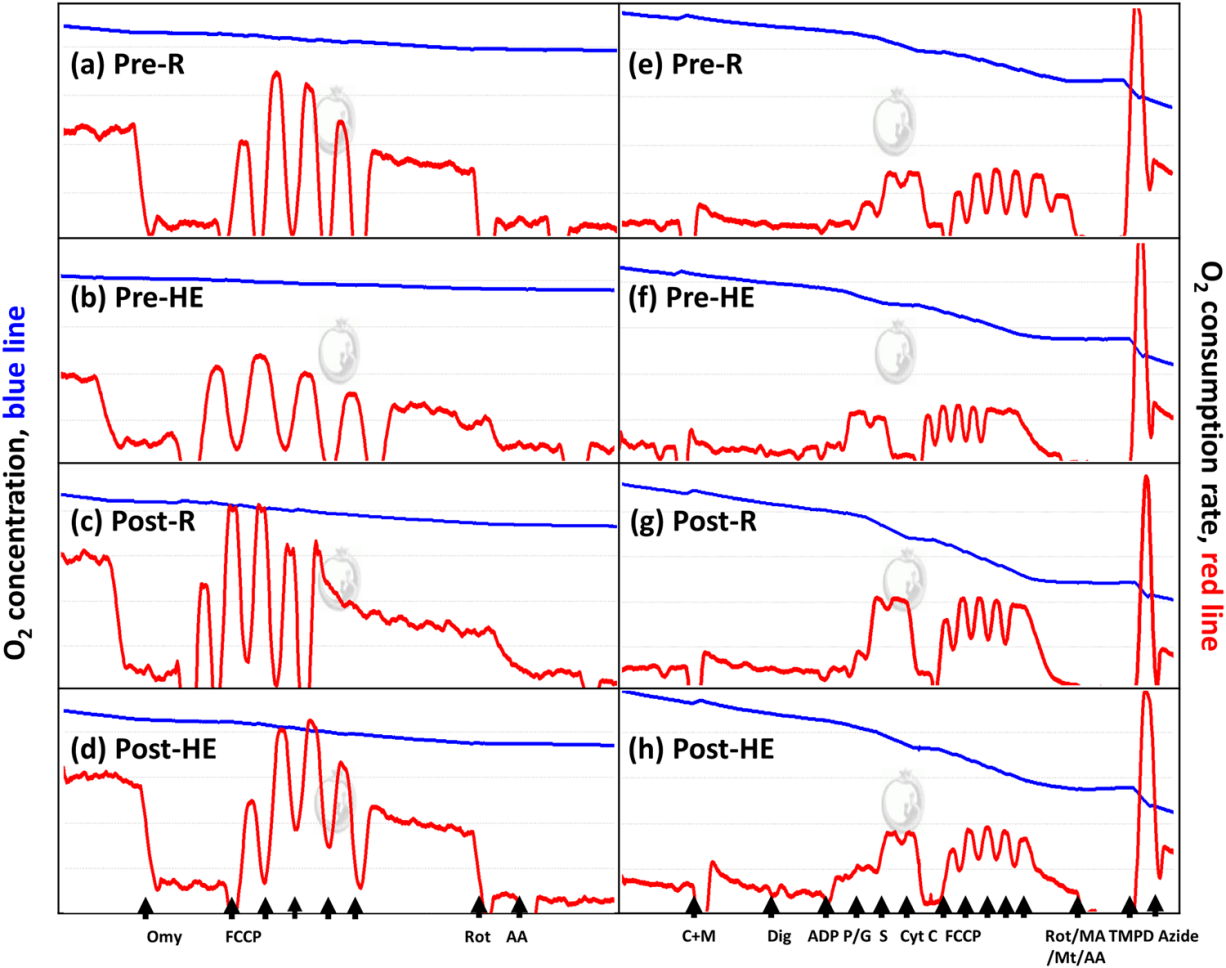
Graph showing measurement of mitochondrial O_2_ consumption rate (OCR) in platelets using a high-resolution respirometry (Oroboros O2K). **(a–d)**, the OCR protocol in intact platelets; **(e–h)**, the OCR [substrate, uncoupler, inhibitor titration (SUIT)] protocol in permeabilized platelets. **Omy**, oligomycin; **FCCP**, carbonyl cyanide-p-trifluoromethoxyphenylhydrazone; **Rot**, rotenone; **AA**, antimycin A; **M**, malate; **C**, palmitoyl-DL carnitine-HCl; **Dig**, digitonin; **ADP**, adenosine diphosphate; **P**, pyruvate; **G**, glutamate; **S**, succinate; **Cyst C**, cytochrome c; **MA**, malonic acid; **Mt**, myxothiazol. **Pre**, before high-intensity interval training (HIT); **Post**, after HIT; **R**, resting; **HE**, hypoxic (12%O_2_) exercise test.

**Figure 4:**
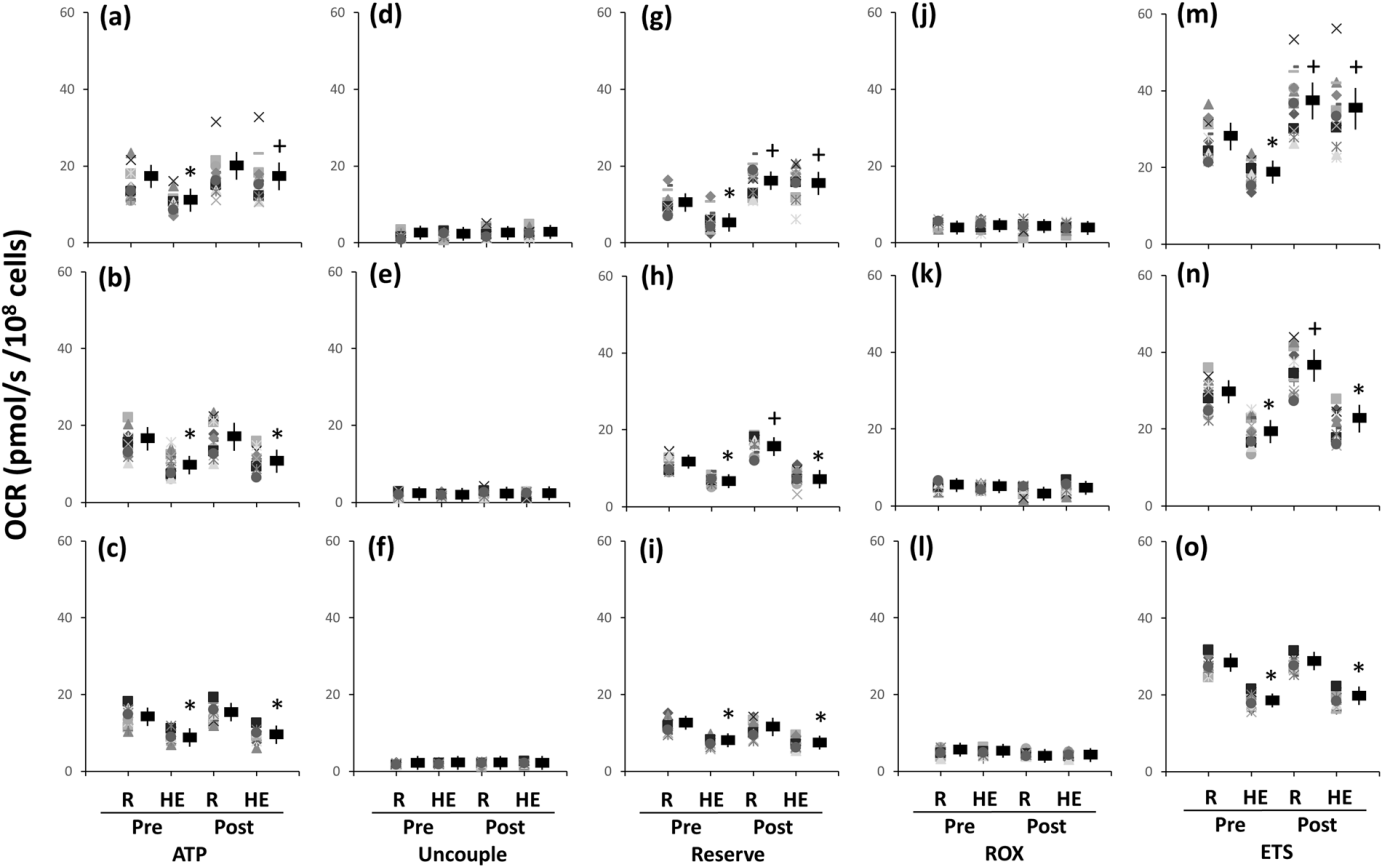
Effects of interval and continuous exercise regimens on mitochondrial O_2_ consumption rate (OCR) in the intact platelets. **HIT (a**,**d**,**g**,**j**,**m)**, high-intensity interval training group; **MCT (b**,**e**,**h**,**k**, **n)**, moderate-intensity continuous training; **CTL (c**,**f**,**i**,**l**,**o)**, control group; **Pre**, pre-intervention; **Post**, post-intervention; **R**, resting; **HE**, hypoxic (12%O_2_) exercise test; **ATP**, ATP-linked OCR; **Uncouple**, uncouple OCR; **RESERVE**, the reserve capacity of OCR; **ROX**, residual O_2_ consumption. * *P* < 0.05, **R** vs. **HE**; ^+^ *P* < 0.05, **Pre** vs. **Post**. Values were mean ± SEM.

**Figure 5:**
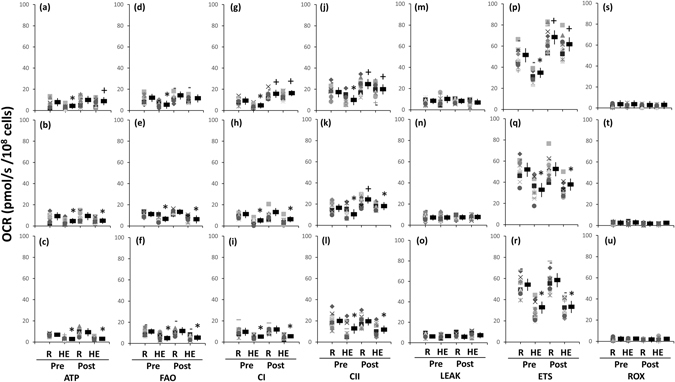
Effects of interval and continuous exercise regimens on mitochondrial O_2_ consumption rate (OCR) in the permeabilized platelets. **HIT (a**,**d**,**g**,**j**,**m**,**p**,**s)**, high-intensity interval training group; **MCT (b**,**e**,**h**,**k**,**n**,**q**,**t)**, moderate-intensity continuous training; **CTL (c**,**f**,**i**,**l**,**o**,**r**,**u)**, control group; **Pre**, pre-intervention; **Post**, post-intervention; **R**, resting; **HE**, hypoxic (12%O_2_) exercise test; **ATP**, ATP-linked OCR; **FAO**, fatty acid oxidation; **ETS**, electron transport system; **CI**, Complex I respiration; **CII**, Complex I respiration; **ROX**, Residual O_2_ consumption. * *P* < 0.05, **Rt** vs. **HE**; ^+^ *P* < 0.05, **Pre** vs. **Post**. Values were mean ± SEM.

Figures 3a–h show the analysis of O_2_ consumption rate (OCR) in the intact (3a–3d) and permeabilized (3e–3h) platelets using high-resolution respirometry (Oroboros O2K). An acute bout of 12%O_2_ exercise significantly decreased ATP-linked OCR (Fig. 4a–c, *P* < 0.05) and the reserve capacity of OCR (Fig. 4g–i, *P* < 0.05) in intact platelets. The HE also suppressed the fatty acid oxidation (FAO)- (Fig. 5d–f, *P* < 0.05), Complex I- (Fig. 5g–i, *P* < 0.05), and Complex II- (Fig. 5j–l, *P* < 0.05) mediated OCRs in permeabilized platelets. After 6 weeks, both HIT and MCT significantly elevated the reserve capacity of OCR in intact platelets at rest (Fig. 4g and h, *P* < 0.05), whereas only HIT alleviated the suppression of the OCR reserve capacity caused by HE (Fig. 4g, *P* < 0.05). Moreover, the two exercise regimens enhanced the Complex II-mediated OCR at rest (Fig. 5j and k, *P* < 0.05), while only HIT diminished the depression of FAO- (Fig. 5d), Complex I- (Fig. 5g), and Complex II- (Fig. 5j) mediated OCRs and the capacity for electron transport system (ETS) (Fig. 5p) in platelets caused by HE. However, there were no significant changes in the HE-mediated mitochondrial respiration of platelets after 6 weeks of CTL (Figs 4 and 5).

**Figure 6:**
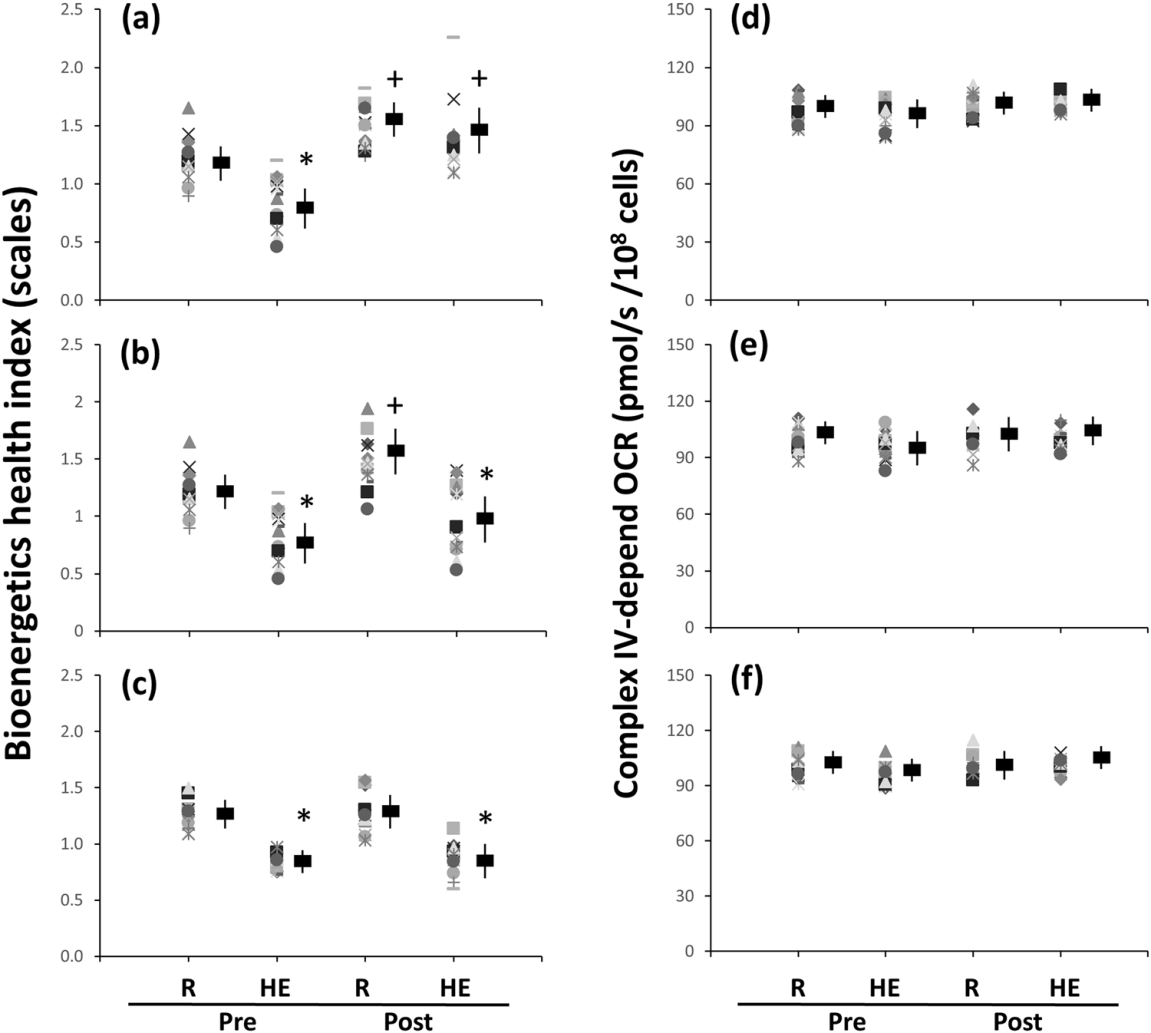
Effects of interval and continuous exercise regimens on **(a–c**) bioenergetics health index (BHI) and **(d–f)** Complex IV activity in platelets. **HIT**, high-intensity interval training group **(a**,**d)**; **MCT**, moderate-intensity continuous training **(b**,**e)**; **CTL**, control group **(c**,**f)**; **Pre**, pre-intervention; **Post**, post-intervention; **R**, resting; **HE**, hypoxic (12%O_2_) exercise test. * *P* < 0.05, **R** vs. **HE**; ^+^ *P* < 0.05, **Pre** vs. **Post**. Values were mean ± SEM.

Before various interventions, the HE test also suppressed the bioenergetics health index (BHI) value in platelets (Fig. 6a–c, *P* < 0.05). After 6 weeks, both HIT (Fig. 6a, *P* < 0.05) and MCT (Fig. 6b, *P* < 0.05) significantly elevated the BHI value in platelets at rest, whereas only HIT attenuated the suppression of BHI caused by HE (Fig. 6a, *P* < 0.05). However, the activities of Complex IV in platelet mitochondria at rest or after HE remained unchanged following 6 weeks of HIT, MCT, or CTL (Fig. 6d–f).

### Enzyme activities of glycolysis and the Krebs cycle in platelets

Acute HE increased the activity of lactate dehydrogenase (LDH) and decreased the activities of citrate synthase (CS) and succinate dehydrogenase (SDH) in platelets (Table 3, *P* < 0.05). After 6 weeks of the intervention, HIT, but not MCT, attenuated the extents of the HE-enhanced LDH activity and the HE-depressed CS and SDH activities in platelets (Table 3). However, the two exercise regimens did not influence the activities of hexokinase (HK) and pyruvate dehydrogenase (PDH) in platelets at rest and after HE (Table 3). Additionally, various enzyme activities of glycolysis and Krebs cycle in platelets at rest or after HE remained unchanged after 6 weeks of CTL (Table 3).

**Table 3:** The effects of interval and continuous exercise regimens on enzyme activities of glycolysis and Krebs cycle in platelets.

	HIT		MCT		CTL	
	Pre	Post	Pre	Post	Pre	Post
***Glycolysis***						
Hexokinase activity (nmol/min/10^8^ cells)						
R	27.9 ± 1.9	26.7 ± 1.7	24.7 ± 2.4	25.9 ± 1.1	24.4 ± 1.4	23.3 ± 1.3
HE	27.5 ± 1.7	27.3 ± 1.1	24.8 ± 2.7	26.6 ± 1.5	25.7 ± 1.2	23.8 ± 1.1
Pyruvate dehydrogenase activity (nmol/min/10^8^ cells)						
R	1.18 ± 0.03	1.13 ± 0.03	1.16 ± 0.03	1.04 ± 0.03	1.26 ± 0.05	1.18 ± 0.04
HE	1.30 ± 0.04	1.17 ± 0.03	1.27 ± 0.04	1.12 ± 0.03	1.26 ± 0.06	1.26 ± 0.05
Lactate dehydrogenase activity (nmol/min/10^8^ cells)						
R	32.2 ± 4.5	30.1 ± 1.6	31.6 ± 2.5	31.8 ± 2.5	35.6 ± 2.4	33.5 ± 2.6
HE	40.1 ± 4.6*	33.6 ± 1.5^+^	41.2 ± 2.4*	37.9 ± 2.5*	42.5 ± 2.2*	41.6 ± 2.8*
***Krebs cycle***						
Citrate synthase activity (nmol/min/10^8^ cells)						
R	5.50 ± 0.22	5.83 ± 0.25	5.63 ± 0.17	5.74 ± 0.21	5.54 ± 0.17	5.68 ± 0.34
HE	4.45 ± 0.32*	5.88 ± 0.43^+^	4.61 ± 0.24*	4.61 ± 0.32*	4.52 ± 0.24*	4.55 ± 0.29*
Succinate dehydrogenase activity (nmol/min/10^8^ cells)						
R	9.46 ± 0.82	9.59 ± 1.32	9.37 ± 0.91	9.62 ± 0.83	9.12 ± 0.42	9.41 ± 0.45
HE	6.77 ± 0.91*	8.82 ± 0.81^+^	6.39 ± 0.92*	6.83 ± 0.72*	6.47 ± 0.61*	6.57 ± 0.60*

Values were mean ± SEM. HIT, high-intensity interval training group; MCT, moderate-intensity continuous training group; CTL, control group; Pre, pre-intervention; Post, post-intervention; R, resting; HE, hypoxic exercise test. *P < 0.05, R vs. HE; ^+^ *P* < 0.05, *P*re vs. Post.

### Dynamic TG in platelet-rich plasma (PRP)

**Figure 7:**
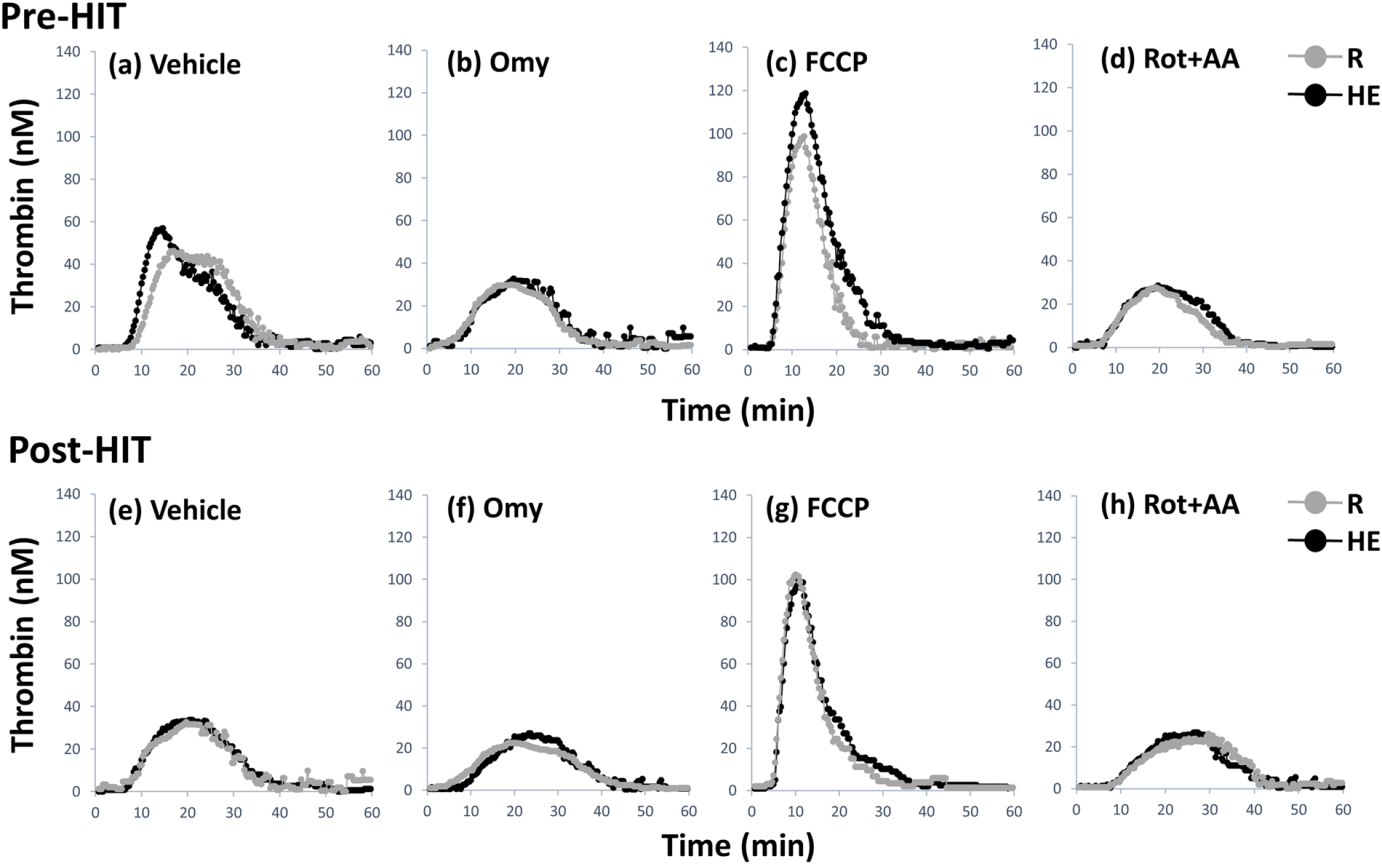
The calibrated, automatic thrombinographic analysis of high-intensity interval training (**HIT**) effect on dynamic thrombin generation in platelet-rich plasma (PRP). **Pre-HIT**, before HIT; **Post-HIT**, after HIT; **R**, resting; **HE**, hypoxic (12%O_2_) exercise test; **Omy**, oligomycin; **FCCP**, carbonyl cyanide-p-trifluoromethoxyphenylhydrazone; **Rot**, rotenone; **AA**, antimycin A.

**Figure 8:**
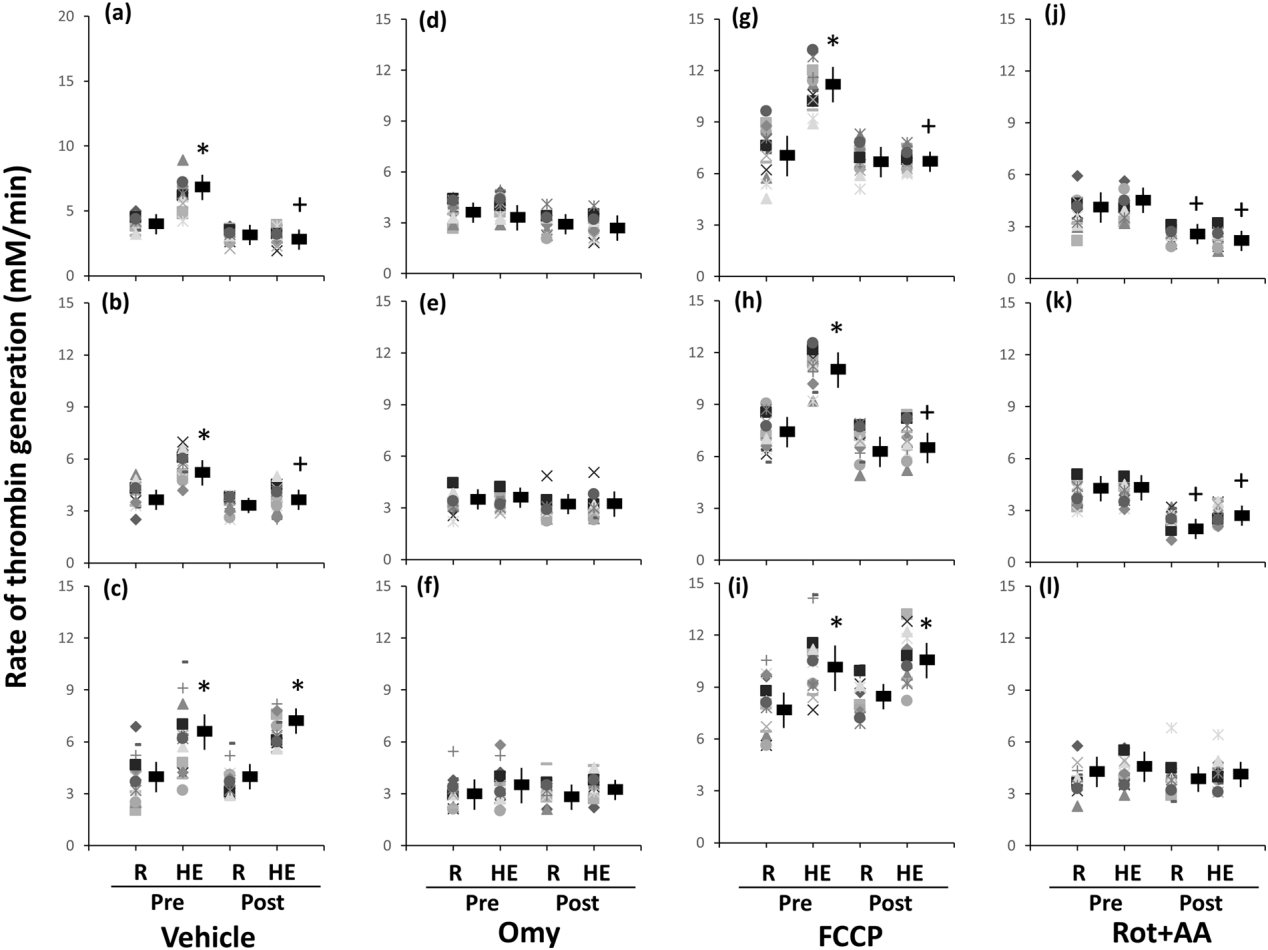
Effects of interval and continuous exercise regimens on the rate of thrombin generation in platelet-rich plasma. **HIT** (**a**,**d**,**g**,**j)**, high-intensity interval training group; **MCT (b**,**e**,**h**,**k**), moderate-intensity continuous training; **CTL** (**c**,**f**,**i**,**l)**, control group; **Pre**, pre-intervention; **Post**, post-intervention; **R**, resting; **HE**, hypoxic (12%O_2_) exercise test; **ETP**, endogenous thrombin potential; **Omy**, oligomycin; **FCCP**, carbonyl cyanide-p-trifluoromethoxyphenylhydrazone; **Rot**, rotenone; **AA**, antimycin A. * *P* < 0.05, **R** vs. **HE**; ^+^ *P* < 0.05, **Pre** vs. **Post**. Values were mean ± SEM.

Figure 7a–g show the samples of the HE-mediated dynamic TG in pretreating PRP with various mitochondrial modulators following 6 weeks of HIT. With respect to the analytic parameters of dynamic TG, acute HE increased the ETP (Table 3, *P* < 0.05), peak height (Table 4, *P* < 0.05), and rate (Fig. 8a–c, *P* < 0.05) of TG but not changed the lag time of TG (Table 4) in PRP before the intervention. Moreover, the changes of parameters of dynamic TG caused by HE were attenuated by pretreating PRP with oligomycin (Table 4 and Fig. 8d–f) or rotenone plus antimycin A (Table 4 and Fig. 8j–l). Six weeks of HIT (Fig. 7a–d) and MCT (Fig. 7E–H) significantly decreased the extents of HE-promoted ETP (Table 4, *P* < 0.05) and peak height (Table 4, *P* < 0.05) and rate (Fig. 8a, *P* < 0.05) of TG in PRP. However, pretreating PRP with oligomycin (Table 4, Fig. 8d–f), rather than rotenone plus antimycin A (Table 4 and Fig. 8j–l), inhibited the training effects of HIT and MCT on the dynamic TG in PRP. Additionally, CLT did not significantly change the values of resting and HE-mediated dynamic TG parameters in untreated and oligomycin- or rotenone plus antimycin A-treated PRP (Table 4 and Fig. 8).

**Table 4:** The effects of interval and continuous exercise regimens on dynamic thrombin generation in platelet-rich plasma.

	HIT		MCT		CTL	
	Pre	Post	Pre	Post	Pre	Post
***Vehicle***						
Lag time (min)						
R	9.1 ± 0.1	9.0 ± 0.1	9.1 ± 0.2	8.9 ± 0.1	9.1 ± 0.2	9.1 ± 0.2
HE	9.1 ± 0.1	9.0 ± 0.1	9.2 ± 0.1	8.9 ± 0.1	9.2 ± 0.2	9.3 ± 0.2
ETP (mM × min)						
R	879 ± 25	876 ± 24	873 ± 14	871 ± 15	855 ± 25	885 ± 25
HE	1105 ± 34*	845 ± 6^+^	1101 ± 35*	870 ± 14^+^	1103 ± 42*	1139 ± 48*
Peak height (mM)						
R	50.7 ± 1.7	45.5 ± 1.2	52.2 ± 1.3	49.6 ± 1.4	50.3 ± 3.7	51.0 ± 1.2
HE	70.1 ± 3.2*	45.0 ± 1.3^+^	67.6 ± 2.5*	49.1 ± 1.7^+^	71.6 ± 5.1*	76.3 ± 2.9*
***Oligomycin***						
Lag time (min)						
R	8.9 ± 0.1	9.0 ± 0.1	8.9 ± 0.1	8.8 ± 0.1	8.8 ± 0.2	8.7 ± 0.2
HE	8.8 ± 0.1	8.9 ± 0.1	8.8 ± 0.1	8.7 ± 0.1	8.8 ± 0.2	8.9 ± 02
ETP (mM × min)						
R	779 ± 31	795 ± 17	791 ± 17	791 ± 14	795 ± 25	782 ± 20
HE	825 ± 32	788 ± 29	845 ± 18	803 ± 17	846 ± 25	834 ± 41
Peak height (mM)						
R	45.0 ± 1.2	44.7 ± 1.6	45.3 ± 1.2	44.3 ± 1.6	44.2 ± 3.0	43.7 ± 1.2
HE	47.0 ± 1.3	44.0 ± 1.5	46.8 ± 1.2	44.0 ± 1.4	46.2 ± 3.0	44.9 ± 1.0
***Carbonyl cyanide-p-trifluoromethoxyphenylhydrazone***						
Lag time (min)						
R	6.9 ± 0.2	7.0 ± 0.1	6.9 ± 0.2	7.0 ± 0.1	7.0 ± 0.3	6.9 ± 0.2
HE	6.7 ± 0.2	7.0 ± 0.1	6.9 ± 0.2	6.9 ± 0.1	6.9 ± 0.2	7.1 ± 0.2
ETP (mM × min)						
R	1005 ± 23	955 ± 22	1027 ± 25	1032 ± 21	1069 ± 28	1040 ± 26
HE	1222 ± 28*	951 ± 20^+^	1215 ± 32*	1025 ± 18^+^	1252 ± 30*	1243 ± 33*
Peak height (mM)						
R	78.1 ± 3.4	79.2 ± 2.9	82.6 ± 2.8	76.8 ± 2.9	83.6 ± 6.1	87.3 ± 2.1
HE	108.1 ± 3.4*	78.6 ± 1.7^+^	106.5 ± 2.7*	79.4 ± 2.62^+^	111.7 ± 7.2*	112.5 ± 4.8*
***Rotenone plus Antimycin A***						
Lag time (min)						
R	9.0 ± 0.2	9.0 ± 0.1	9.1 ± 0.2	8.8 ± 0.1	9.0 ± 0.2	8.7 ± 0.2
HE	8.9 ± 0.2	8.9 ± 0.1	9.0 ± 0.1	8.8 ± 0.1	9.0 ± 0.2	8.8 ± 0.3
ETP (mM × min)						
R	833 ± 22	723 ± 21^+^	840 ± 31	749 ± 16^+^	833 ± 20	829 ± 26
HE	847 ± 20	735 ± 21^+^	879 ± 27	754 ± 13^+^	859 ± 24	841 ± 24
Peak height (mM)						
Rest	49.0 ± 2.1	33.3 ± 0.9^+^	45.3 ± 1.8	34.7 ± 1.3^+^	46.4 ± 3.0	47.0 ± 1.3
HE	50.4 ± 1.7	32.6 ± 1.0^+^	48.1 ± 1.5	37.0 ± 1.8^+^	49.8 ± 3.1	47.8 ± 1.5

Values were mean ± SEM. HIT, high-intensity interval training group; MCT, moderate-intensity continuous training group; CTL, control group; Pre, pre-intervention; Post, post-intervention; R, resting; HE, hypoxic exercise test; ETP, endogenous thrombin potential. *P < 0.05, R vs. HE; ^+^ *P* < 0.05, *P*re vs. Post.

## Discussion

Mitochondrial dysfunction is associated with oxidative stress and thought to be a common underlying mechanism of metabolic and cardiovascular diseases^14–16^. This study is the first to demonstrate that acute bout of 12% O_2_ exercise elevates mitochondrial oxidative stress and subsequently suppresses the CS and SDH activities and the ETS efficiency in platelets, which responses are accompanied by accelerating TG in PRP. Notably, the HIT regimen effectively ameliorates the HE-induced mitochondrial dysfunction of platelets by depressing the cellular oxidative stress, which may reduce the risk of platelet-mediated TG evoked by hypoxia.

### Mitochondrial oxidative phosphorylation in platelets

Mitochondria are highly sensitive to hypoxic stress and respond dynamically to changes in their cellular microenvironment^17^. The present study showed that acute HE decreased mitochondrial MP and increased MOB in platelets, which may reflect mitochondrial oxidative damage of platelets caused by HE. Additionally, the HE-induced decrease in ATP-linked OCR and reserve capacity of OCR may be associated with modified activities of mitochondrial enzymes and/or an impeded the flow of electrons, thereby decreasing the ETS efficiency after HE. Acute HE also significantly decreased the CS and SDH activities and was accompanied by increased the LDH activity, suggesting that the HE shifts platelet metabolic pathways from mitochondrial respiration to glycolysis^18, 19^. Furthermore, the HE substantially depressed the capacity of Complex I- and II-related respirations and the efficiency of ETS in platelets in a substrate-rich environment. Summarily, acute HE globally decreases mitochondrial substrate availability and impairs mitochondrial bioenergetics and/or integrity in platelets.

In this investigation, the HIT regimen significantly enhanced platelet CS and SDH activities, as well as, Complex I- and II-related respirations following HE. The enhanced OCR of platelets by HIT may indicate a greater capacity of mitochondrial OXPHOS in platelets due to increased Complex protein activities of the ETS, elevated ATP production rate, or heightened levels of NADH and FADH_2_. Moreover, increased resistance to the HE-depressed platelet mitochondrial OCR after HIT may improve the flexibility of platelet activation caused by HE. A recent study has indicated that hypoxia or ischemia results in accumulation of intracellular succinate levels, thus leading to elevated mitochondrial ROS production^20^. The elevation of oxidative stress may also induce succinate accumulation by decreasing SDH activity^21^. Therefore, the increased SDH activity and Complex II respiration of platelets in the HIT groups may quickly eliminate succinate, thereby further retarding the ROS production from platelet mitochondria following HE.

### Mitochondrial biogenesis in platelets

Early studies on exercise intervention predominantly focused on mitochondrial functionality in skeletal muscles^22^. An acute bout of exercise promotes transcriptional or post-translational regulation of peroxisome proliferator-activated receptor gamma coactivator-1α, whereas chronic exercise increases the rates of muscular mtDNA gene expression by upregulating Tfam in skeletal muscles^22^. In the present study, no significant changes in resting and HE-related platelet biogenetic parameters, such as the Complex IV/II ratio, were observed after 6 weeks of HIT or MCT. Accordingly, we propose that platelet metabolic adaptation induced by HIT may be associated with improved ETS efficiency rather than modulated mitochondrial biogenesis in platelets.

### Dynamic TG in PRP

Extremely hypoxic environments are associated with increased incidence of vascular thromboemoblic events^23, 24^. Blood is subjected to oxidative stress during extremely hypoxic exposure^4^; elevated oxidative stress may contribute to coagulation system^4^ and platelet activation^11^. In the present study, acute HE facilitated TG in PRP and was accompanied by lost mitochondrial MP and increased MOB in platelets. Hence, increased ROS level by changed coupling of mitochondrial ETS may participate in platelet-mediated TG undergoing hypoxic stress. However, HIT for 6 week markedly depresses the HE-induced oxidative damage of platelet mitochondria and consequently attenuates the platelet-mediated TG caused by HE.

### Limitations of study

Our small size in each group (n = 15) is a major limitation of this study. However, the results of aerobic capacity and mitochondrial bioenergetics and dynamic TG in platelets obtained from this investigation have high values of statistical power from 0.853 to 1.000. Additionally, the subjects used tended to be young, males, and healthy, thus further clinical evidence was required to extrapolate the present results to the elderly, females or patients with platelet or thrombotic disorders.

In this study, acute bout of HE promoted dynamic TG in PRP, whereas pretreating PRP with mitochondrial ETS inhibitors attenuated acute exercise effects. Moreover, 6 weeks of HIT also suppressed the extent of HE-promoted dynamic TG in PRP. Whether the suppression of HE-induced TG in PRP caused by HIT involves (i) change of plasma composition or/and (ii) down-regulation of procoagulant factors on platelets to diminish the enhancement of plasma TG by HE remains unresolved. Hence, the potential confounding effects of the different plasma compositions at various experimental conditions need further study.

Aim of this study mainly focus on examining the effects of exercise on platelet-induced TG rather than platelet reactivity (adhesion and aggregation). Our previous studies have investigated the effect of exercise training on platelet adhesiveness and aggregability and their underling mechanisms^25^. However, the role of platelet mitochondrial function on exercise-mediated platelet reactivity requires further explored.

### Conclusions

Acute 12% O_2_ exercise elevated oxidative stress and decreased the ETS efficiency in platelet mitochondria. Although no changes were found in mitochondrial biogenesis, 6 week of HIT enhanced mitochondrial OXPHOS capacity and attenuated the HE-depressed mitochondrial ETS efficiency in platelets. Therefore, the HIT regimen effectively improve platelet bioenergetics, possibly by enhancing mitochondrial quality rather than quantity in platelets. Moreover, HIT also markedly reduces the enhancement of platelet-mediated TG by HE, which response is associated with alleviating the HE-induced mitochondrial dysfunction of platelets. These experimental findings may facilitate the identification of effective exercise training regimens to increase aerobic capacity and minimize platelet mitochondrial dysfunction and subsequent thrombus formation evoked by hypoxic stress.

## Materials and Methods

### Subjects

The study was in accordance with the Declaration of Helsinki and approved by the Chang Gung Memorial Hospital Institutional Review Board, Taiwan. All experiments were performed in accordance with relevant guidelines and regulations. A total of 45 sedentary males who were non-smokers, did not use medications or vitamins, and were free of any cardiopulmonary/hematological risks were recruited from Chang Gung University, Taiwan. According to our previous studies, the menstrual phases of women influence activity of cardiac autonomic nervous system^26^ and platelet reactivity^27^. Hence, this study excluded female subjects to avoid the effects of gender and the menstrual phase on cardiovascular^26^ and hematological^27^ functions. No subjects had engaged in any regular physical activity (exercise frequency ≤ once weekly, duration < 20 min) or had been exposed to high altitudes (≥altitude of 3000 m) for at least 1 year before the experiment. All subjects provided informed consent after the experimental procedures were explained. These subjects were randomly divided into three groups: the HIT (n = 15), MCT (n = 15), and CTL (n = 15) groups. Moreover, all subjects arrived at the testing center at 9:00 AM to eliminate any possible circadian effects. The experimental environment was maintained at a temperature of 22+/− 0.5 °C with a relative humidity of 60+/− 5%. Participants were instructed to fast for at least 8 hours and to refrain from strenuous physical exercise for at least 48 hours before sampling.

### Training protocols

Both the HIT and MCT groups performed exercise regimens on a stationary bicycle ergometer 5 times a week for 6 weeks. For comparison, CTL participants did not any undergo any exercise but were carefully monitored, and we recorded information on their physical activity and nutritional intake for 6 weeks. HIT subjects warmed up for 3 min at 30% of VO_2max_ before starting five exercise cycles, each lasting 3 min at 80% of VO_2max_ interspersed with a 3 min active recovery period at 40% of VO_2max_. The exercise session was terminated with a 3 min cool-down period at 30% of VO_2max_. The MCT group had the same warm-up and cool-down protocols as the HIT group, except that the training period was 30 min at 60% of VO_2max_^13, 28^. The two exercise protocols were isovolumic with the same exercise duration [i.e., HIT exercise volume: 6 min (40%VO_2max_ + 80%VO_2max_) × 5 cycles = MCT exercise volume: 30 min (60%VO_2max_)]. Each subject used a heart rate (HR) monitor (Tango, SunTech Medical) to obtain the assigned intensity of exercise. The work-rate of the bicycle ergometer was adjusted continuously to ensure that the intensity of exercise matched the target HR throughout the training period. The percentage of HR reserve (%HRR) is widely considered to be equivalent to the percentage of VO_2_ reverse for exercise prescription purposes^29^. Accordingly, the target HRs of HIT and MCT were calculated using the following equations^29^:

1 $${\rm{Peak}}\,{\rm{HR}}=220-{\rm{age}}$$

2 $$\begin{array}{c} \% {\rm{HHR}}= \% ({\rm{peak}}\,{\rm{HR}}-{\rm{resting}}\,{\rm{HR}})+{\rm{resting}}\,{\rm{HR}}\end{array}$$

3 $${\rm{Target}}\,{\rm{HR}}\,{\rm{of}}\,{\rm{HIT}}=3-{\rm{minute}}\,{\rm{intervals}}\,{\rm{at}}\,40 \% \,{\rm{HHR}}\,{\rm{and}}\,80 \% \,{\rm{HRR}}$$

4 $${\rm{Target}}\,{\rm{HR}}\,{\rm{of}}\,{\rm{MCT}}={\rm{sustained}}\,60 \% \,{\rm{HRR}}$$

All subjects recorded their daily activities and nutritional intake throughout the experiment using the International Physical Activity Questionnaire Short Form^30^ and the Written Diet Record^31^, respectively. The participants were instructed to refrain from extra regular exercise until the end of the study. Moreover, the participant compliance rates for the three interventions were 100%. All subjects completed the exercise intervention and/or tests at the beginning of the present study and after 6 weeks in the three groups.

### GXT

Subjects performed a GXT on a bicycle ergometer (Corival 400, Lode) to assess their aerobic capacity 4 days before and 4 days after the 6 week interventions^18, 28^. The GXT was composed of 2 min of unloaded pedaling followed by a continuous increase in work-rate of 30 W per 3 min until exhaustion (*i*.*e*., VO_2max_). The V_E_, VO_2_, and VCO_2_ were measured breath by breath with a computer-based system (MasterScreen CPX, Cardinal-health Germany). The defined VO_2max_ was required to achieve the following 3 of 4 criteria: (i) the level of VO_2_ increased less than 2 mL/kg/min over at least 2 min; (ii) HR exceeded its predicted maximum; (iii) the respiratory exchange ratio exceeded 1.2, and (iv) the venous lactate concentration exceeded 8 mM, consistently with the guidelines of the American College of Sports Medicine for exercise testing^32^. The value of ventilatory threshold was determined by applying the following ventilatory criteria; (i) the V_E_/VO_2_ ratio increased without a corresponding increase in the V_E_/VCO_2_ ratio; (ii) PETO_2_ increased without a decrease in the PETCO_2_, and (iii) a departure from linearity for V_E_^32^.

### The HE test and blood collection

Each subject performed the HE test on the 2^nd^ day before the intervention and on the 2^nd^ day after the intervention in an air-conditioned normobaric hypoxia chamber (Colorado Mountain Room, Boulder, CO) as described in our previous studies^18, 28^. The hypoxia chamber was maintained at a temperature of 22+/− 0.5 °C with a relative humidity of 60+/− 5%; a CO_2_ scrubber eliminated CO_2_ in the air (<3,500 ppm). The HE test on the bicycle ergometer required 50 W of warm-up for 3 min, an increase in the work-rate to 100 W with continuous exercise for 30 min, and then recovery to 50 W of a cool-down period for 3 min. During the test, the O_2_ concentration was set to 12%, which corresponds to an altitude of 4,460 m^19, 29^. For safety reasons, the HE test was terminated immediately when the level of O_2_ saturation dropped to <70% or the subject complained of discomfort. All subjects were free of acute mountain sickness symptoms during the experimental period.

At rest and immediately after the HE test, 40 ml blood samples were collected from an antecubital vein using a clean venipuncture (20 gauge needle). The first 2 ml of blood was discarded, and the remaining blood was used to measure hematological parameters. Blood cells were counted using a Sysmax SF-3000 cell counter (GMI, Inc., Ramsey, MN).

### Platelet isolation

Blood samples were collected in polypropylene tubes that contained sodium citrate (3.8 g/dl, 1–9 vol. blood). PRP was prepared by centrifugation at 300 *g* for 10 min at room temperature (20 °C). Platelets were pelleted by centrifugation of the PRP at 1,500 *g* for 10 min and then washed once with phosphate buffered saline containing ethylenediaminetetraacetic acid (final concentration, 4 mM) (Sigma) to prohibit platelet activation. The number of platelets was adjusted to 2 × 10^8^ cells/ml with RPMI medium (Sigma). The tests of blood samples were repeated to ensure reproducibility. The analysis of platelet functions was completed within 2 hours after cell purification.

### Mitochondrial content, MP, and MOB in platelets

The relative quantification of platelet mitochondrial-localized dyes using the single-color flow cytometric analysis allows for the sensitive measurement of a variety of mitochondrial parameters, including mitochondrial content, MP, and MOB, as described in previous studies^18, 33^. The platelet suspensions (2 × 10^8^ cells/ml) were incubated with MitoTracker Green FM (a green-fluorescent mitochondrial stain, 200 nM) (Invitrogen), tetramethylrhodamine ethyl ester (TMRE, a mitochondrial potential-sensitive probe, 20 nM) (Invitrogen), or MitoSOX Red (a mitochondrial superoxide indicator, 6.6 μM) (Invitrogen) in the dark for 30 min at 4 °C. Then, the platelets were gated separately from other particles on the basis of forward/sideways scatter, and the mean fluorescence intensity from 100,000 events representing the platelets was calculated using a FACScan flow cytometer (Becton Dickinson).

### Mitochondrial respiration and BHI in intact platelets

The mitochondrial O_2_ consumption of platelets (2 × 10^8^ cells/ml) in RPMI 1460 medium was measured using high-resolution respirometry (Oroboros O2K). Mitochondrial respiration coupled towards ATP production (ATP-linked OCR) was measured by the fall in O_2_ consumption following the addition of oligomycin (0.2 μg/ml), an inhibitor of ATP synthase. The remaining rate of mitochondrial respiration represents a proton leak that uncouples OXPHOS from the ETS. The total O_2_ consumption of platelets was measured at baseline and after the addition of the uncoupling agent carbonyl cyanide-p-trifluoromethoxyphenylhydrazone (FCCP; 2 μM) to induce maximal O_2_ consumption. The difference between the basal and maximal respiration is called the reserve capacity of OCR. Non-mitochondrial respiration (non-mito OCR) was quantified by inhibiting mitochondrial respiration through the addition of rotenone (a mitochondrial Complex I inhibitor, 1 μM) and antimycin A (a mitochondrial Complex III inhibitor, 1 μM)^18, 34^.

The BHI was calculating from the result of coupling control protocol to quantify platelet mitochondrial function using the following equations^35^:

1 $$\mathrm{Log}[(\mathrm{ATP}-\mathrm{linked}\,{\rm{OCR}})\times ({\rm{Reserved}}\,{\rm{capacity}}\,{\rm{of}}\,{\rm{OCR}})/({\rm{Proton}}\,{\rm{leak}})\times (\mathrm{Non}-\mathrm{mito}\,{\rm{OCR}})]$$

2 $$\mathrm{ATP}-\mathrm{linked}\,{\rm{OCR}}={\rm{Routine}}\,{\rm{state}}-{\rm{Leak}}\,{\rm{state}}$$

3 $${\rm{Reserved}}\,{\rm{capacity}}={\rm{ETS}}\,{\rm{state}}-{\rm{Routine}}\,{\rm{state}}$$

4 $$\mathrm{Non}-\mathrm{mito}\,{\rm{OCR}}={\rm{Residual}}\,{{\rm{O}}}_{2}{\rm{consumption}}\,({\rm{ROX}}){\rm{state}}$$

5 $${\rm{Proton}}\,{\rm{leak}}={\rm{Leak}}\,{\rm{state}}-{\rm{ROX}}\,{\rm{state}}$$

### Mitochondrial respiration in permeablized platelets

A substrate, uncoupler, inhibitor titration (SUIT) protocol was applied to permeablized platelets preparation and analyzed the respiration capacity of platelet mitochondria. By multiple substrate titration, electron flow from FAO and mitochondrial Complex I and II were well controlled, and the mitochondrial OCR of each state was measured by a high-resolution respirometry (Oroboros O2K)^18, 34^.

Intact platelets (2 × 10^8^ cells/ml) were incubate at 37 °C in the O2K chamber of mitochondria respiratory medium MiR05 (EGTA 0.5 mM, MgCl_2_ · 6H_2_O 3 mM, lactobionic acid 60 mM, taurine 20 mM, KH_2_PO_4_ 10 mM, HEPES 20 mM, D-sucrose 110 mM, and bovine serum albumin 1 g/l, pH = 7.1). Data acquisition was started after the oxygen flux stabilized. The O_2_ consumption in this state was the routine respiration from endogenous substrates in cells. Then the plasma membrane was permeabilized by digitonin (0.1 mg) titration after a concomitant addition of malate (2 mM) and palmitoyl-DL-carnitine-HCl (20 μM). Since the absence of adenylate in the chamber, the respiration in this state was caused by mitochondrial proton leakage (LEAK). The O_2_ consumption by FAO was evaluated by addition of 1 mM ADP (Calbiochem). Oxidative phosphorylation capacity of mitochondrial Complex I and II were acquired through the addition of NADH resources pyruvate (5 mM) and glutamate (10 mM) and SDH resource succinate (10 mM). A cytochrome c (10 μM) test was applied to evaluate whether the outer mitochondrial membrane was intact. The maximal convergent capacity of the ETS was subsequently obtained by FCCP titration (0.5 μM/steps).

Finally, the inhibitors for Complex I, II and III (0.1 μM rotenone, 5 mM malonic acid, and 0.5 μM myxothiazol/2.5 μM antimycin A) were progressively added to suppress the O_2_ consumption of platelets mitochondria. At last, tetramethyl-p-phenylenediamine (TMPD, 0.5 mM) applied as an artificial substrate for reducing cytochrome c, was added for cytochrome c oxidase activity measurement and then blocked by mitochondrial Complex IV inhibitor sodium azide (200 mM). All chemicals were purchased from Sigma-Aldrich (St Louis, MO, USA) if not stated otherwise.

### Enzyme activities of glycolysis and the Krebs cycle in platelets

The activities of HK (Sigma), PDH (BioVision), and LDH (Sigma) in glycolysis and the activities of CS (BioVision) and SDH (BioVision) in the Krebs cycle of platelets (1 × 10^8^ cells/ml) were measured with commercially available colorimetric kits according to the manufacturer’s instructions^18^.

### Dynamic TG assay

The dynamic TG in PRP was measured by calibrated, automatic thrombinography (Synapse/Thrombinoscope BV, Maastricht, the Netherlands), as described in our earlier works^4, 13^. Oligomycin (2 μg/ml), FCCP (3.75 μM), or rotenone (0.1 μM) plus antimycin A, (2.5 μM) were added to the PRP (2 × 10^8^ cells/ml), which was then warmed to 37 °C for 10 min. Following incubation, eighty microliters of the PRP samples were allocated into the wells of round bottom 96-well microtiter plates (Nunc). Twenty microliters of reagent containing tissue factor (TF) was mixed with the plasma samples to obtain a final concentration of 0.5 pM TF. Coagulation was started by adding 0.1 M CaCl_2_ (20 μL) in a fresh mixture of fluobuffer (containing 20 mM HEPES and 60 mg/mL bovine serum albumin in pH 7.35) containing 2.5 mM Z-Gly-Gly-Arg-AMC (the fluorogenic substrate) (Synapse/Thrombinoscope BV, Maastricht, the Netherlands). Upon cleaving by thrombin, the fluorescent AMC (7-amino-4-methylcoumarin) is released and measured with a 390-nm-excitation and a 460-nm-emission filter set in an Ascent Fluoroskan (Thermo Fisher Scientific Inc., the Netherlands). All reagents were warmed to 37 °C before the experiment began. Fluorescence was recorded for 60 min. The fluorescence signal was corrected for substrate consumption, plasma colour variability and inner filter fluorescence effect by running in parallel calibrating wells where 80 microliters plasma samples were mixed with 20 microliters Thrombin Calibrator from Thrombinoscope BV.

The calculated data using analytic software from Synapse/Thrombinoscope BV were plotted and expressed in terms of lag time (time until initial thrombin formation, min), endogenous thrombin potential (ETP, area under the thrombin curve, nM × min), the peak height of thrombin (nM), and the rate of TG (mean slope = peak height/[time to peak-lag time], nM/min)^4, 13^.

### Statistical analysis

The results are expressed as the mean ± SEM. The statistical software package StatView was used for data analysis. Experimental results in each group were analyzed by the repeated measures ANOVA and Bonferroni’s post-hoc test to compare the count, mitochondrial OXPHOS, oxidative stress and biogenesis of platelets, as well as, the dynamic TG in PRP before and immediately after HE at the beginning of the present study and after 6 weeks. In addition, the comparison of cardiopulmonary fitness during GXT at the beginning of the present study and 6 weeks later in various groups was analyzed by the repeated measures ANOVA and Bonferroni’s post-hoc test. The criterion for statistical significance was *P* < 0.05.
